# Effect of Bio-Inspired Polymer Types on Engineering Characteristics of Cement Composites

**DOI:** 10.3390/polym14091808

**Published:** 2022-04-28

**Authors:** Se-Jin Choi, Sung-Ho Bae, Jae-In Lee, Eun Ji Bang, Hoe Young Choi, Haye Min Ko

**Affiliations:** 1 Department of Architectural Engineering, Wonkwang University, 460 Iksan-daero, Iksan 54538, Korea; csj2378@wku.ac.kr (S.-J.C.); caos1344@naver.com (S.-H.B.); wodls103@naver.com (J.-I.L.); 2 Department of Chemistry, Wonkwang University, 460 Iksan-daero, Iksan 54538, Korea; dmswlal1@naver.com (E.J.B.); wonderlost1412@naver.com (H.Y.C.); 3 Department of Chemistry, Wonkwang Institute of Material Science and Technology, Wonkwang University, 460 Iksan-daero, Iksan 54538, Korea

**Keywords:** bio-inspired polymer, chitosan, cement composite, compressive strength, durability

## Abstract

Cement concrete is the most commonly used building and construction material worldwide because of its many advantages. Over time, however, it develops cracks due to shrinkage and tension, which may lead to premature failure of the entire structure. Recently, the incorporation of polymers has been explored to improve the overall strength and durability of cement concrete. In this study, two types of chitosan-based bio-inspired polymers (a-BIP and b-BIP) were synthesized and mixed with cement mortar in different proportions (5–20%). The fluidity of the resulting mixtures and the properties of the hardened samples, such as the compressive and tensile strengths, drying shrinkage, and carbonation resistance, were evaluated. The characteristics of the polymers were tuned by varying the pH during their syntheses, and their structures were characterized using nuclear magnetic resonance spectroscopy, Fourier-transform infrared spectroscopy, and ultraviolet-visible spectroscopy. After 28 days of aging, all samples containing BIPs (35.9–41.4 MPa) had noticeably higher compressive strength than the control sample (33.2 MPa). The tensile strength showed a similar improvement (up to 19.1%). Overall, the mechanical properties and durability of the samples were separately dependent on the type and amount of BIP.

## 1. Introduction

Cement concrete has been widely used as the primary construction material because of its easy workability, high durability, and fire resistance. However, it has low tensile strength and is susceptible to cracking and structural deterioration due to external forces, chemical reactions, and volume changes [1,2,3]. Recently, increasing maintenance costs due to the increase in the number of aged concrete structures have generated interest in improving the durability and safety of concrete structures [4,5,6]. In this regard, several studies have revealed that admixtures [7,8,9] and functional polymer materials [10,11,12,13,14,15,16,17,18] are useful for improving the performance of mortar or concrete.

When a polymer is mixed with cement mortar and concrete, it improves the strength, impact resistance, waterproofness, corrosion resistance, and adhesion strength [15,19,20,21,22,23,24,25,26]. Heidarnenezhad et al. [23] reported increases in compressive strength and tensile strength of lightweight polymer concrete with increasing polymer ratio. Lokuge et al. [24] studied the effect of the resin type on the mechanical properties of polymer concrete and reported that polymer-based filler materials are effective in improving the compressive and tensile strengths. Niaki et al. [25] evaluated the mechanical properties of epoxy-based polymer concrete using basalt fibers and clay nanoparticles, and reported that these components improved the mechanical properties and thermal stability of polymer concrete.

Wang et al. [26] studied the strength characteristics of epoxy-based polymer concrete using crumb rubber and reported improved compressive strength and tensile strength of the sample mixed with 5% solid rubber. In addition, there have been some recent studies on cement mortar or concrete using bio-inspired polymer (BIP) materials [10,27]. Choi et al. [10] synthesized a catechol-functionalized chitosan (Cat-Chit) polymer that improved the compressive strength when used with cement mortar. Mohesson et al. [27] studied the properties of concrete when mixed with biopolymer alginate, which effectively improved the compressive and tensile strengths.

Chitosan, obtained from the deacetylation of chitin extracted from the exoskeleton of crustaceans such as lobster, crab, and shrimp, is a unique non-toxic biopolymer consisting of β-(1,4)-2-acetamido-2-deoxy-D-glucose and β-(1,4)-2-amino-2-deoxy-D-glucose units. The numerous hydroxyl and amine groups in chitosan chains induce strong intramolecular hydrogen bonding in solution. Additionally, the hydrophobic moieties, such as the acetyl group and glucoside ring in chitosan, tend to cause aggregation via hydrophobic interactions [28,29,30]. Chitosan has also received significant attention due to its potential antibacterial, anti-tumor, and anti-microbial properties [31,32,33,34,35,36]. However, despite being a promising biomaterial, the use of chitosan is limited by its low solubility in water. Therefore, many research groups have reported a synthetic method for preparing chitosan conjugated with catechol groups, which are widely found in marine mussels, to improve the solubility and adhesion. The discovery of this approach has contributed to various biomedical applications of chitosan, such as antibacterial coatings [37,38], tissue adhesives [39], and drug delivery platforms [40]. Thus, as a BIP, catechol-conjugated chitosan, synthesized via a general amide coupling reaction, could be a promising alternative for enhancing the performance of cement mortar.

In this study, two types of BIPs were synthesized by varying the pH and were mixed with cement mortar in different ratios. The performance of these cement mortar samples was evaluated in terms of flow, compressive strength, tensile strength, drying shrinkage, and carbonation resistance.

## 2. Materials and Methods

### 2.1. Materials

Chitosan (medium molecular weight; degree of deacetylation: 75–85%) and hydrocaffeic acid (HCA; 3-(3,4-dihydroxyphenyl)propionic acid) were purchased from Sigma-Aldrich. 1-Ethyl-3-(3-dimethylaminopropyl)carbodiimide hydrochloride (EDC) and ethanol were purchased from Tokyo Chemical Industry (Tokyo, Japan) and Fisher Scientific (Waltham, MA, USA), respectively. All the chemicals were of analytical grade and were used without further purification. A membrane with a molecular weight cut-off of 12–14 kDa (Spectra/Por, Spectrum Laboratories, Irving, TX, USA) was used for dialysis. Ultrapure water (Millipore, Burlington, MA, USA) was used in all experiments.

For the mortar, ordinary Portland cement (specific gravity: 3.15 g/cm^3^; Blaine: 3430 cm^2^/g; Asia company, Seoul, Korea) and fine aggregates (sand from the Namwon region; specific gravity: 2.6 g/cm^3^, Fineness modulus: 2.89) were used. 

### 2.2. Synthesis of BIP

The type A BIP (a-BIP) was synthesized according to a reported procedure [41,42]. Typically, chitosan (500 mg) was dissolved in distilled water (DW, 50 mL), and the solution pH was adjusted to 5.5 using 1.0 M HCl. Solutions of HCA (500 mg) in ethanol/DW (10 mL/10 mL) and EDC (500 mg) in ethanol/DW (2.5 mL/2.5 mL) were added dropwise to the chitosan solution. The pH was maintained at 5.5 using 1.0 M HCl while the reaction mixture was stirred at room temperature (24 °C) for 12 h. After reaction completion, the mixture was dialyzed in 0.1 M NaCl solution (NaCl 30 g/4.5 L DW), whose pH was maintained at 5.0 using 1.0 M HCl for 48 h and then in DW (4.5 L) for 4 h. Finally, the solution was frozen at −20 °C and lyophilized (−75 °C/5 mTorr). 

The type B BIP (b-BIP) was synthesized according to a previously reported procedure [43,44]. First, chitosan (500 mg) was dissolved in a mixture of 5 N HCl (2.5 mL) and DW (23 mL). Subsequently, solutions of HCA (590 mg) in ethanol (2.5 mL) and EDC (625 mg) in ethanol (10 mL) were slowly added to the chitosan solution. The pH of the resultant mixture was adjusted to 5.0 using a saturated NaOH solution, and the reaction mixture was stirred at room temperature for 12 h. Next, the pH of the mixture was again adjusted to 5.0 using 5 N HCl solution. The mixture was dialyzed in a 0.01 M NaCl solution for 48 h and then in DW for 24 h, with a change of the dialysate every 6 h. Finally, the solution was frozen at −20 °C and lyophilized (−75 °C/5 mTorr). 

The absorbance of the aqueous BIP solution was measured using a UV–vis spectrophotometer (GENESYS 180, Thermo Fisher, Waltham, MA, USA). To obtain proton nuclear magnetic resonance (^1^H-NMR) spectra (500 MHz, JEOL, Tokyo, Japan), the BIP samples were dissolved in D_2_O. Fourier-transform infrared (FT-IR) (Nicolet iS5, Thermo Fisher Scientific) spectra were obtained in the wavenumber range of 400–4000 cm^−1^ at a resolution of 1 cm^−1^.

### 2.3. Mixing Proportions and Specimen Preparation

For all cement mortar samples in this study, the water–cement (W/C) ratio was fixed at 50%, and water, cement, and sand contents were 170, 340, and 739 kg/m^3^, respectively. Solutions of BIPs (500 mg of a-BIP or b-BIP dissolved in 1000 mL of water) were prepared and added in proportions of 5, 10, 15, and 20.0% with respect to the amount of the mixing water, as indicated by suffixes on the sample labels, e.g., a-BIP05 contained 5% a-BIP. For comparison, a control sample without either BIP was also prepared. Table 1 shows the mix proportion used in this study.

A mechanical mixer for mortar mixing with a stainless steel bowl was used to mix the raw materials. Immediately after the preparation of the sample mixtures, changes in pH (HI 8314, HANNA instruments, USA) were recorded. Subsequently, cubic specimens (dimensions 50 mm × 50 mm × 50 mm) and cylindrical specimens (∅50 mm × 100 mm) were prepared by molding for compressive strength and split-tensile strength tests, respectively. Additional specimens (dimensions 40 mm × 40 mm × 160 mm) were prepared for ultrasonic pulse velocity (Pundit, Proceq, Zurich, Switzerland), drying shrinkage, and accelerated carbonation tests. Each specimen was demolded after 24 h and cured in water at 20 °C. The flow of the mortar mixtures and compressive strength of the samples were measured according to the KS L 5105 standard [45], while their tensile strengths and drying shrinkages were measured according to the KS F 2423 [46] and KS F 2424 [47] standards, respectively. The presented strength values are the average values of three samples.

The accelerated carbonation test was performed in an accelerated carbonation chamber (at a constant temperature of 20 ± 2 °C, constant humidity of 60 ± 5%, and constant CO_2_ concentration of 5 ± 0.2%) according to the KS F 2584 standard [48], in which the carbonation depth was measured using phenolphthalein solution.

Scanning electron microscopy (SEM) (AIS1800C, SERON Technologies, Seoul, Korea) images were obtained to analyze the morphology of the samples.

## 3. Results and Discussion

### 3.1. Characteristics of BIP

**Figure 1:**
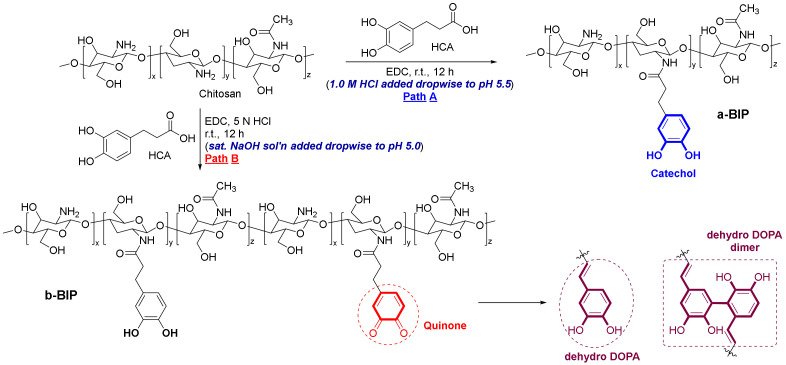
Synthetic scheme for BIPs under different pH conditions.

The catechol group of HCA is a well-known organic moiety, which is pH-sensitive and can be easily oxidized to its highly reactive quinone form at a pH above 5.5. The quinone spontaneously undergoes irreversible polymerization to form dicatechol dimers and covalent crosslinking networks [49,50,51,52]. Therefore, controlling the pH during the reaction is crucial for preparing polymers with exact functional groups. The BIPs were synthesized by the EDC-mediated amide coupling reaction of chitosan and HCA under slightly different acidic conditions (Figure 1). Interestingly, the final products depend on the methods used to maintain the pH near 5.0, such as the dropwise addition of the acid (Figure 1, Path A) or base (Figure 1, Path B) solutions. Due to the susceptibility of catechol toward oxidation, covalently crosslinked b-BIP was obtained via path B using a saturated NaOH solution, whereas a-BIP with complete catechol was obtained via path A using HCl solution. 

**Figure 2:**
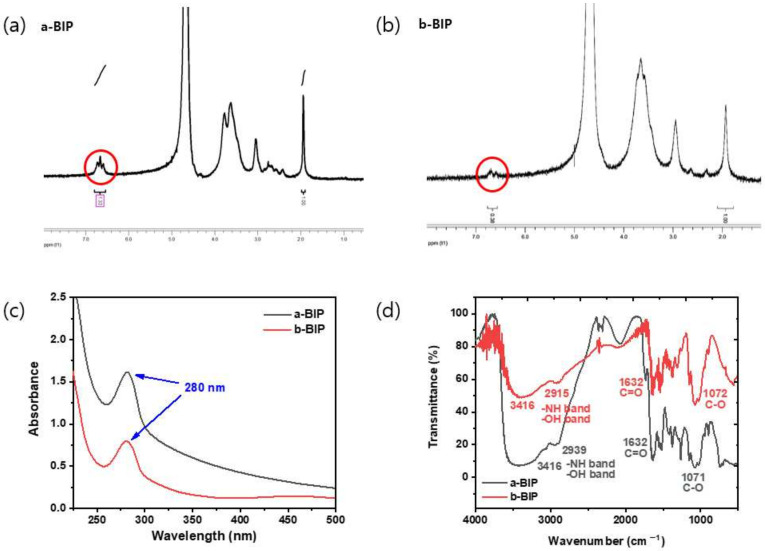
Qualitative analysis of BIPs: ^1^H-NMR spectra of (**a**) a-BIP and (**b**) b-BIP; (**c**) UV–vis spectra of a-BIP (black line) and b-BIP (red line); (**d**) FT-IR spectra of a-BIP (black line) and b-BIP (red line).

The degree of catechol conjugation (DOC_cat_) in the BIPs was calculated by ^1^H NMR spectroscopy from the ratio of the peak integrals for catechol protons to those for the acetyl group (Figure 2a,b). The results showed that ~27% and ~7% of the total amino groups were transformed into amides (attached with 3,4-dihydroxyhydrocinnamic acid groups) in a-BIP and b-BIP, respectively. Similar DOC_cat_ values of ~29% and ~4% were calculated for a-BIP and b-BIP, respectively, from the absorption peak of catechol at 280 nm using UV—vis spectroscopy with HCA as the standard molecule (Figure 2c). Finally, FT-IR spectroscopic analysis was performed to identify specific functional groups (Figure 2d). In both polymers, the hydroxyl and amino groups were represented by the peak at approximately 3416 cm^−1^, whereas the carbonyl groups of amides were represented by the peak at approximately 1632 cm^−1^.

### 3.2. Fluidity and pH of Cement Mortars

**Figure 3:**
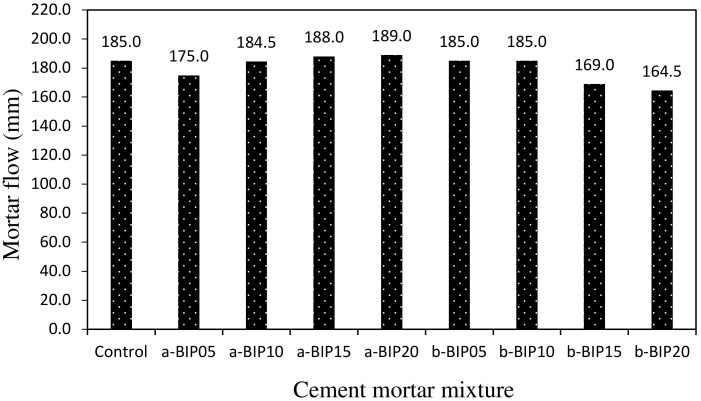
Mortar flow values of different cement mortar mixtures.

Figure 3 shows the variation in mortar flow according to the type and amount of BIP used. For the mixtures containing a-BIP, the flow increased with increasing BIP amount, whereas for those containing b-BIP, the opposite trend was observed. The flow of the b-BIP20 sample was ~164.5 mm, which is relatively low. The flow of the control sample was ~185 mm, similar to those of the samples containing 10–20% a-BIP and 5–10% b-BIP. It has been reported previously [10] that the use of BIPs does not affect the flow of cement mortar significantly. However, in this study, when more than 15% b-BIP was used, the mortar flow was reduced by ~9–11%.

**Figure 4:**
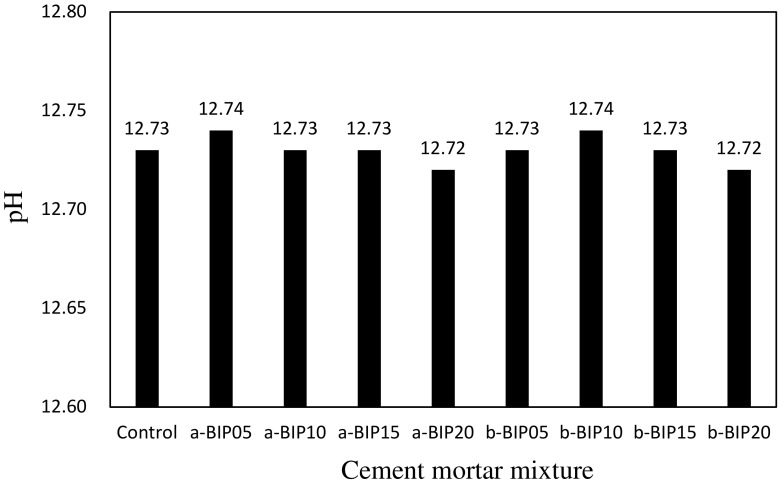
pH levels of different cement mortar mixtures.

Figure 4 shows the change in the pH of the cement mortar mixture samples according to the type and amount of BIP used. The pH of the a-BIP20 and b-BIP20 mixtures was 12.72, which was slightly lower than that of the control sample. Irrespective of the type and amount of BIP used, all the samples exhibited similar alkalinities with pH levels in the range of 12.72–12.74. Therefore, it was concluded that the type and amount of BIP do not affect the pH of the cement mortar significantly.

### 3.3. Compressive Strength

**Figure 5:**
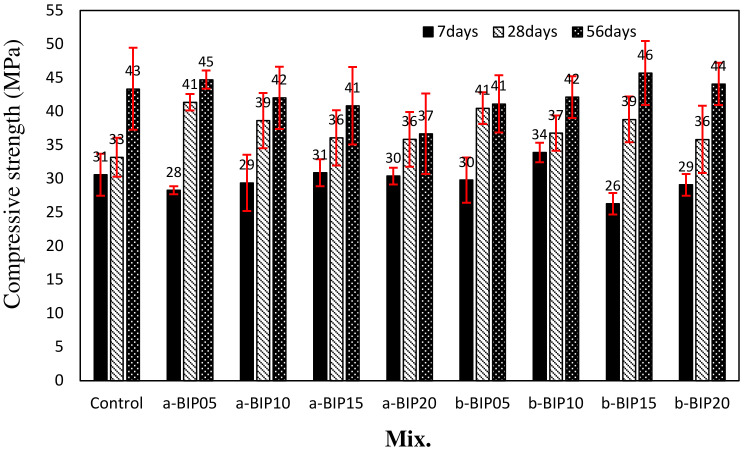
Compressive strengths of different cement mortar samples.

Figure 5 shows the change in the compressive strength of different cement mortar samples with aging time. After 7 days of aging, the compressive strength of the control sample was ~30.6 MPa, whereas those of the samples containing a-BIP were in the range of 28.3–30.9 MPa. The compressive strengths of the a-BIP15 and a-BIP20 samples were in the range of ~30.4–30.9 MPa, comparable to that of the control sample. The 7-day compressive strengths of the samples containing b-BIP were in the range of ~26.3–33.9 MPa, with b-BIP10 exhibiting a compressive strength of 33.9 MPa, ~10.7% higher than that of the control sample.

After 28 days of aging, the compressive strength of the control sample increased to ~33.2 MPa, whereas the strengths of all the BIP-containing samples were in the range of ~35.9–41.4 MPa. The 28-day compressive strength of the a-BIP samples was approximately 8.1–24.6% higher than that of the control sample. In particular, the a-BIP05 sample exhibited the highest compressive strength of ~41.4 MPa. It has been reported [10] that the system with calcium–BIP crosslinking or chelating complexes afforded a higher compressive strength than the control system with no BIP. The 28-day compressive strengths of the b-BIP samples were in the range of ~35.9–40.5 MPa, which were approximately 8.1–21.9% higher than that of the control sample. Likewise, the compressive strength of the b-BIP05 sample was the highest. The compressive strength tended to decrease with increasing a-BIP amount. This could be attributed to the self-aggregation effect when a large amount of polymer is used [10]. Compared to the a-BIP samples, the difference between the compressive strengths of the b-BIP samples was smaller.

After 56 days of aging, there was a negligible increase in the strengths of the a-BIP05 and b-BIP05 samples. In contrast, samples with a low 28-day compressive strength showed a significant increase in compressive strength. Among the a-BIP samples, the a-BIP05 sample exhibited the highest 56-day compressive strength of ~44.7 MPa. Among the b-BIP samples, the b-BIP15 sample exhibited the highest 56-day compressive strength (~45.7 MPa).

### 3.4. Ultrasonic Pulse Velocity

**Figure 6:**
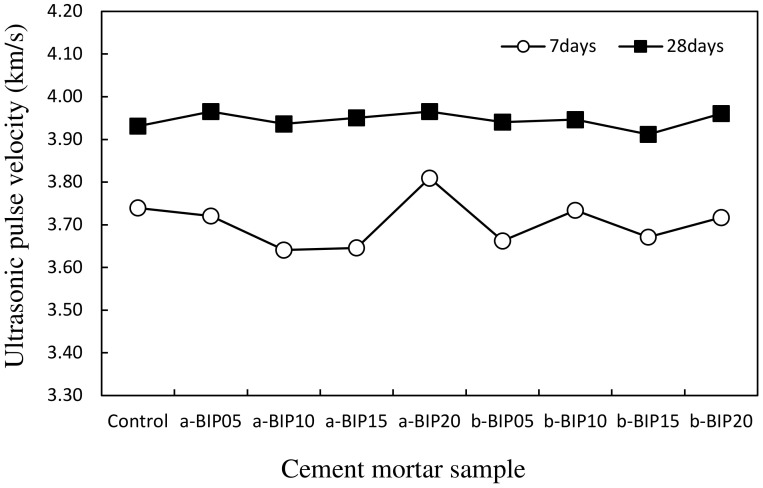
Ultrasonic pulse velocities across the cement mortar samples.

Figure 6 shows the ultrasonic pulse velocities across the different cement mortar samples. The velocity range for the samples was 3.64–3.81 km/s after 7 days of aging, and increased slightly to 3.91–3.97 km/s after 28 days, suggesting densification of the cement matrix as the hydration reaction of cement progressed. Although the velocity across the 28-day samples was approximately 4.1–7.4% higher than that across the 7-day samples, there was no clear difference among the samples in terms of the BIP type or content.

### 3.5. Split-Tensile Strength

**Figure 7:**
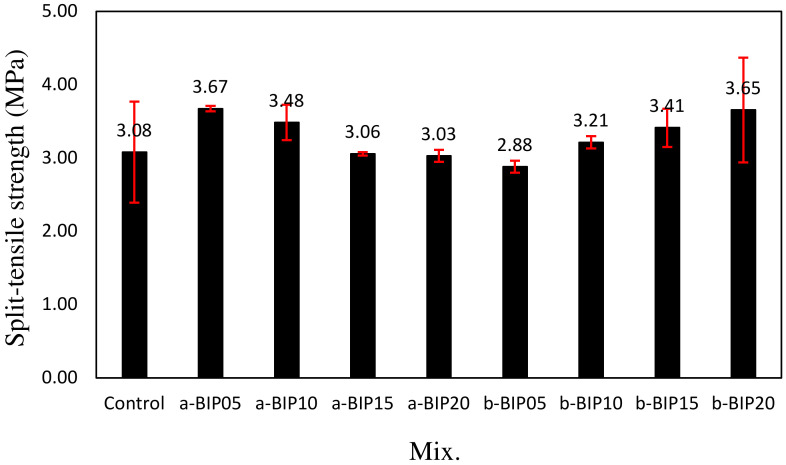
Split-tensile strengths of different cement mortar samples after 28 days of aging.

Figure 7 shows the tensile strengths of different cement mortar samples after 28 days of aging. Similar to the compressive strength, the tensile strengths of the samples containing BIPs were relatively higher than that of the control sample. Interestingly, the tensile strength of the samples containing a-BIP decreased with increasing BIP amount, whereas those of the samples containing b-BIP showed the opposite trend. The highest tensile strengths were exhibited by a-BIP05 (3.67 MPa) and b-BIP20 (3.65 MPa), which were approximately 18.5% and 19.1% higher than that of the control sample, respectively.

### 3.6. Drying Shrinkage

**Figure 8:**
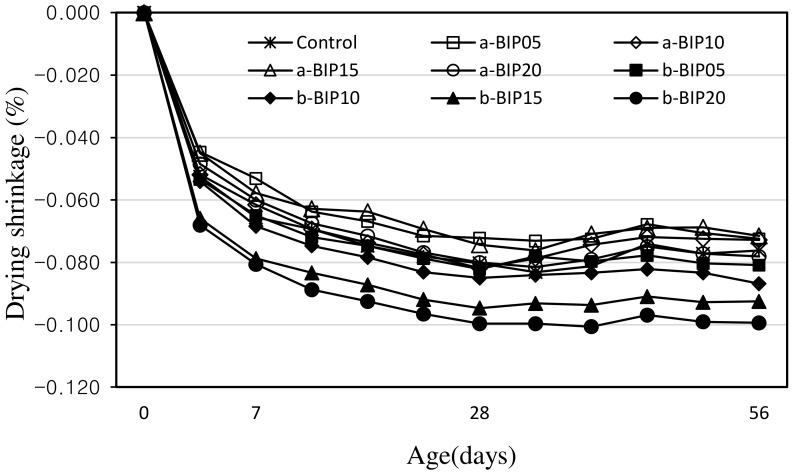
Drying shrinkages in different cement mortar samples.

Figure 8 shows the change in the drying shrinkage of the cement mortar samples according to the aging time. After 56 days, drying shrinkage in the samples containing a-BIP was in the range of ~0.072–0.078%, which was lower than that in the control sample (~0.076%) except for a-BIP20 (0.078%). In contrast, the drying shrinkage in the samples containing b-BIP was in the range of ~0.081–0.099%, which was ~6.5–30.2% higher than that in the control sample. Moreover, the drying shrinkage in the samples increased with increasing amount of b-BIP. The shrinkage was more pronounced in b-BIP15 and b-BIP20, especially during the initial aging. However, it stabilized over time, similar to other samples. 

**Figure 9:**
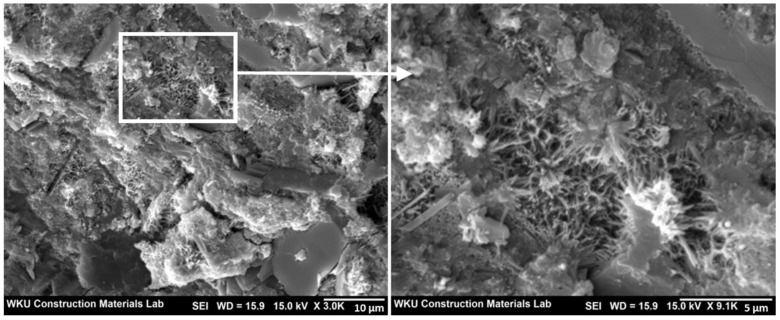
SEM images of b-BIP20 sample after 7 days.

Since the drying shrinkage in these samples was large in the initial age, the drying shrinkage value after 56 days was higher than those of the other samples. The observed large drying shrinkage during initial aging in some samples containing BIP seemed to be due to the aforementioned self-aggregation phenomenon. It is likely that the self-aggregated materials generated due to the use of a large amount of polymer acted as defective regions, thereby reducing the strength and increasing the available space for shrinkage in the cement matrix. This was confirmed in the SEM images (Figure 9) of the 7-day-aged b-BIP20 sample.

### 3.7. Carbonation Resistance

**Figure 10:**
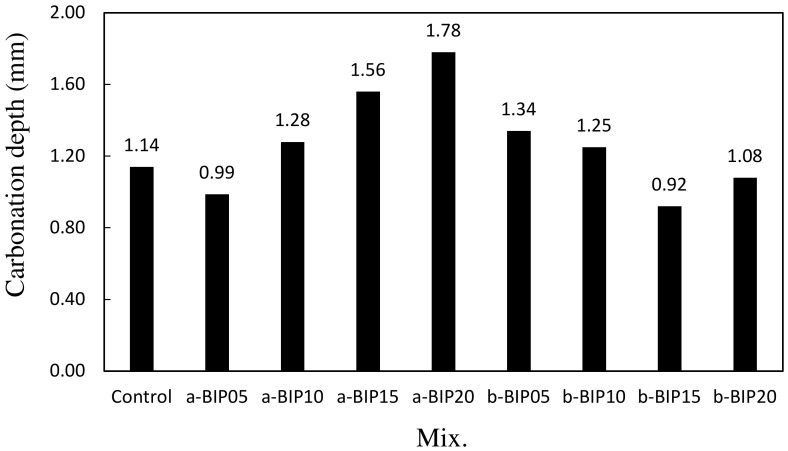
Carbonation depths of different cement mortar samples.

Figure 10 shows the carbonation depth values of the cement mortar samples after 28 days of accelerated carbonation. The carbonation depth of the control sample was ~1.14 mm. When a-BIP was used, the carbonation depth slightly decreased to 0.99 mm (a-BIP05) and then increased with increasing amount of a-BIP. In general, as the compressive strength increases, the cement matrix becomes denser and carbonation depth decreases. When b-BIP was used, the lowest carbonation depth was observed in the b-BIP15 sample (0.92 mm), which also showed the highest compressive strength after 56 days. In contrast, the sample that showed the lowest compressive strength after 56 days, i.e., a-BIP20, showed the largest carbonation depth (~1.78 mm). In this study, a strong correlation was obtained between the 56-day compressive strength and accelerated carbonation depth. 

## 4. Conclusions

The results of this study can be summarized as follows:

1. The structure (non-crosslinked or crosslinked) of the BIPs depended on the pH conditions during the amidation reaction.
2. The introduction of a new functional group, such as catechol and dicatechol dimer, as an additive to polymer backbones could improve the properties of cement mortar.
3. After 28 days of aging, the compressive strength of the control sample was ~33.2 MPa. The incorporation of BIPs improved the compressive strength to the range of ~35.9–41.4 MPa. In particular, the highest compressive strength of ~41.4 MPa was exhibited by the a-BIP05 sample.
4. The tensile strengths of the samples containing the BIPs were relatively higher than that of the control sample, similar to the trend observed for the compressive strength. In general, appropriate use of BIP was shown to be effective in improving the mechanical properties. In this study, the best results were obtained when 5% a-BIP was used.
5. After 56 days, the drying shrinkages of the samples containing a-BIP were in the range of ~0.072–0.078%, lower than that of the control sample (~0.076%), except for the a-BIP20 sample (0.078%). When b-BIP was used, the drying shrinkage increased to ~0.081–0.099%, which was ~6.5–30.2% higher than that of the control sample.
6. After 28 days of accelerated carbonation, the carbonation depth of the control sample was ~1.14 mm, and the carbonation depth inversely correlated to compressive strength. The lowest carbonation depth (0.99 mm), and therefore, the highest compressive strength was obtained in the sample with 5% a-BIP (a-BIP05).

Further studies are needed to establish the relationship between microstructures of cement composites and durability characteristics depending on the presence of BIPs.

## Figures and Tables

**Table 1:** Mix proportions of mortars.

Mix	a-BIP	b-BIP	W/C	Water	Cement	Sand
	(%)	(%)	(%)	(kg/m^3^)	(kg/m^3^)	(kg/m^3^)
Control	-	-	50	170	340	739
a-BIP05	5	-				
a-BIP10	10	-				
a-BIP15	15	-				
a-BIP20	20	-				
b-BIP05	-	5				
b-BIP10	-	10				
b-BIP15	-	15				
b-BIP20	-	20				

## Data Availability

Not applicable.
